# Psychotic experiences and future school performance in childhood: a population‐based cohort study

**DOI:** 10.1111/jcpp.13281

**Published:** 2020-06-19

**Authors:** Lisa R. Steenkamp, Koen Bolhuis, Laura M. E. Blanken, Maartje P. C. M. Luijk, Manon H. J. Hillegers, Steven A. Kushner, Henning Tiemeier

**Affiliations:** ^1^ Department of Child and Adolescent Psychiatry/Psychology Erasmus Medical Centre‐Sophia Children’s Hospital Rotterdam The Netherlands; ^2^ Generation R Study Group Erasmus Medical Centre Rotterdam The Netherlands; ^3^ Department of Psychology, Education and Child Studies Erasmus University Rotterdam Rotterdam The Netherlands; ^4^ Department of Psychiatry Erasmus University Medical Centre Rotterdam The Netherlands; ^5^ Department of Social and Behavioural Sciences Harvard T.H. Chan School of Public Health Boston MA USA

**Keywords:** Psychosis, school performance, intelligence, cognitive impairment, school children

## Abstract

**Background:**

Psychotic experiences are common in childhood and an important risk indicator of adverse mental health outcomes. However, little is known about the association of psychotic experiences with functional outcomes in childhood, particularly regarding school performance. The aim of the present study was to examine whether psychotic experiences were prospectively related to school performance in childhood.

**Methods:**

This study was embedded in the population‐based Generation R Study (*N = *2,362). Psychotic experiences were assessed using self‐reports on hallucinations at age 10 years. School performance was assessed using a standardized national school performance test at age 12 years. We considered the total school performance score, as well as language and mathematics subscales. Analyses were adjusted for sociodemographic characteristics, maternal nonverbal IQ, nonverbal IQ at age 6 years and co‐occurring psychopathology at age 10 years.

**Results:**

Psychotic experiences were prospectively associated with poorer school performance scores (*B* = −0.61, 95% CI [−0.98;−0.25], *p* = .001), as well as poorer language (*B*_percentile rank score_ = −2.00, 95% CI [−3.20;−0.79], *p* = .001) and mathematical ability (*B*_percentile rank score_ = −1.75, 95% CI [−2.99;−0.51], *p = *.006). These associations remained after additional adjustment for nonverbal IQ at age 6 years (*B* = −0.51, 95% CI [−0.86;−0.16], *p* = .005), and co‐occurring internalizing (*B* = −0.40, 95% CI [−0.77;−0.03], *p* = .036) and externalizing problems (*B* = −0.40, 95% CI [−0.75;−0.04], *p* = .029), but not attention problems (*B* = −0.10, 95% CI [−0.47;0.26], *p* = .57).

**Conclusions:**

Children with psychotic experiences had lower school performance scores than their nonaffected peers. The finding was independent of sociodemographic characteristics, intelligence and co‐occurring internalizing and externalizing problems, but not attention problems. This study suggests that psychotic experiences are associated with childhood functional impairments, although the relatively small effects and the role of attention problems warrant further exploration.

## Introduction

Psychotic experiences are common in children and often co‐occur with mental distress and psychopathology (Bolhuis et al., 2018; Kelleher, Connor et al., 2012; Kelleher et al., 2015). They are conceptualized on a psychosis continuum, which ranges from subclinical psychotic experiences in the general population to distressing florid psychotic symptoms in patients with a formal psychotic disorder (van Os & Reininghaus, 2016). The psychosis continuum is supported by evidence of genetic, cognitive and environmental risk factors shared between psychotic experiences and psychotic disorders (Linscott & Van Os, 2013; Zavos et al., 2014). Individuals with psychotic experiences are at increased risk for psychotic and nonpsychotic disorders, as well as adverse outcomes such as suicidality and a lower quality of life (Healy et al., 2019; Kaymaz et al., 2012; van Os & Reininghaus, 2016; Trotta et al., 2019; Yates et al., 2018).

Given that the majority of prospective studies on psychotic experiences have investigated severe adult outcomes, relatively little is known about whether psychotic experiences result in poorer functional outcomes in childhood, especially school performance. Studies in adolescents and adults have shown a negative association between psychotic experiences and educational achievement (Davies, Sullivan, & Zammit, 2018; Unterrassner et al., 2017). To our knowledge, only one study has investigated the relationship between psychotic experiences and school performance in children, which reported that persistent auditory hallucinations were associated with poorer school performance (Bartels‐Velthuis, van de Willige, Jenner, van Os, & Wiersma, 2011). However, this was based on a relatively small number of children without covarying for important confounders, such as ethnic background, maternal education and co‐occurring psychopathology. Since psychotic experiences frequently co‐occur with other psychological problems (Bolhuis et al., 2018; van Os & Reininghaus, 2016; Wigman et al., 2012), adjusting for co‐occurring psychopathology would provide insight into whether the observed associations are specific for psychotic experiences or might be mediated by other problems arising as a consequence of psychotic experiences.

Moreover, it remains unknown whether the association between psychotic experiences and school performance is independent of early childhood intelligence. This is important in the light of evidence suggesting that adolescents with psychotic experiences have impaired cognitive functioning (Kelleher, Clarke, Rawdon, Murphy, & Cannon, 2012). In addition, longitudinal studies demonstrated that children with lower IQ scores were more likely to report psychotic experiences in adolescence (Horwood et al., 2008; Polanczyk et al., 2010). This is consistent with the identification of poor cognitive functioning as a risk factor for psychosis (Khandaker, Barnett, White, & Jones, 2011).

The present study aimed to examine the association between psychotic experiences at age 10 years and school performance at age 12 years, objectively assessed by a national standardized test of school performance. We hypothesized that children who report psychotic experiences have poorer future school performance, including impaired language and mathematical ability. We also tested whether nonverbal IQ at age 6 years and co‐occurring psychopathology at age 10 years accounted for the association between psychotic experiences and school performance.

## Methods

### Design and study population

The present study was embedded in Generation R, a large population‐based prospective cohort from foetal life until adulthood (Kooijman et al., 2016). In the period 2002 to 2006, all pregnant women living in Rotterdam (the Netherlands) were eligible for inclusion and approximately 61% of them were included at baseline (*N* = 9,778). All study procedures were approved by the Medical Ethics Committee of the Erasmus Medical Centre Rotterdam. We obtained written informed consent from all mothers.

**Figure 1:**
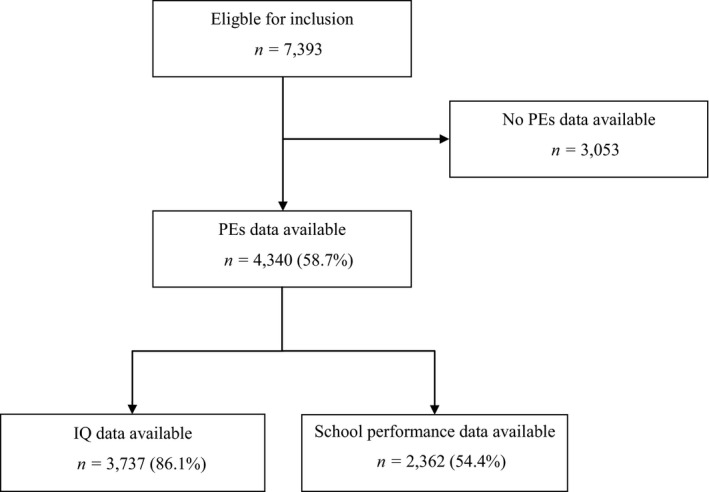
Flowchart inclusion

A flowchart of the study population is shown in Figure 1. Assessment of psychotic experiences was conducted for 4,340 children, and of these children, *n* = 2,362 had data on school performance scores available. The prevalence of psychotic experiences of children in the study population did not differ from the prevalence of psychotic experiences in children not included (*χ^2^ = *4.69, *p* = .10), but included children had higher nonverbal IQ scores (105.2 vs. 101.0, *t* (3524) = −8.80, *p* < .001), were more often of Dutch origin (*χ^2^* = 48.15, *p < *.001) and had mothers with higher educational attainment (*χ^2^* = 31.12, *p < *.001) and higher nonverbal IQ scores (99.8 vs. 97.3, *t* (3675) = −5.57, *p* < .001) than those without school performance data available (*n = *1,978). Nonverbal IQ assessments were available for *n = *3,737 children.

### Psychotic experiences – age 10 years

Two items on auditory and visual hallucinations from the Youth Self‐Report questionnaire were used to assess psychotic experiences at age 10 years (Achenbach & Edelbrock, 1987). These items were selected because they have the highest predictive power of clinician‐confirmed psychotic experiences in young people (Gundersen et al., 2019; Kelleher, Harley, Murtagh, & Cannon, 2009). The items inquired about the presence of hallucinations in the preceding 6 months and were rated on a three‐point scale: not at all (0), a bit (1) or clearly (2). Besides using the sum score of the two items as a continuous variable, we categorized the sum score of psychotic experiences into: no (0 points), mild (a score of 1 point on at least one of the items) and moderate‐to‐severe psychotic experiences (a score of 2 points on at least one of the items). We chose these cut‐offs so that the children in the moderate‐to‐severe category would have ‘clearly’ endorsed at least one of the items (Bolhuis et al., 2018).

### School performance – age 12 years

School performance was assessed with the Dutch standardized end‐of‐primary‐school test, created by the Central Institute for Test Development (CITO; www.cito.com). The CITO test broadly assesses the learning achievement of 12‐year‐old children and is the most widely used academic test in primary school, involving 85% of all Dutch children (van der Lubbe, 2007). CITO test scores range from 501 to 550 points, and each score is traditionally converted to a secondary school recommendation (see Table S1). The CITO test scores are non‐normally distributed (left‐skewed) and have a ceiling effect. Although all the model assumptions were met, we also conducted a sensitivity analysis using the raw CITO test scores. These raw scores are normally distributed but not easily comparable between test years. Hence, we standardized the scores for each test year. In addition, we assessed the relationship between psychotic experiences and school performance using ordinal logistic regression. To this aim, we converted the CITO scores into the corresponding school levels. Since the results of these sensitivity analyses were highly similar to the original results (data available upon request), we decided to report the original CITO test scores to enhance interpretability and to use the full information available in continuous data.

Test scores were obtained from CITO (*n* = 2,028) and, if not available, retrieved from maternal reports by questionnaire (*n* = 334). We assessed inter‐rater reliability between maternal reported and CITO test scores from data linkage (overlap *n* = 975). The intraclass correlation coefficient indicated excellent inter‐rater reliability (ICC = 0.97), supporting the use of maternal‐reported CITO scores as complementary to the test scores obtained from CITO.

We additionally examined the two CITO test subscales: (a) language and (b) mathematics. The language subscale consisted of writing, reading comprehension, spelling, grammar, punctuation and vocabulary. The mathematics subscale comprised dealing with numbers, money and time, mental arithmetic, proportions, fractions and percentages and geometry (Van Boxtel, Engelen, & de Wijs, 2011). Subscale scores were only available for the children with test scores retrieved from CITO (*n = *2,028). Because raw subscale scores are not easily comparable between different CITO test years, we used percentile rank scores as recommended by the publishers.

### Nonverbal IQ – age 6 years

At age 6 years, children were invited to the research centre for the assessment of nonverbal IQ using two subtests of a validated Dutch nonverbal intelligence test ‘the Snijders‐Oomen Niet‐verbale intelligentie Test‐Revisie’ (SON‐R, Tellegen, Winkel, Wijnberg‐Williams, & Laros, 2005). The SON‐R assesses intelligence without being dependent upon language skills and was therefore suitable for our young and multi‐ethnic cohort, in which children of non‐Dutch origin were likely to be less exposed to the Dutch language. The scores were converted into a nonverbal IQ score based on age‐specific reference scores from a Dutch population norm (Tellegen et al., 2005).

### Covariates

Based on previous research, age, sex and ethnicity, and maternal education and IQ were considered to be potential confounders in the relationship between psychotic experiences and school performance (Horwood et al., 2008; Linscott & Van Os, 2013). Children’s ethnicity was determined using the classification procedure of Statistics Netherlands. Ethnicity was defined as Dutch when both parents were born in the Netherlands and Non‐Dutch when at least one parent was born outside of the Netherlands. Non‐Dutch children were classified into Other Western and Non‐Western. Maternal educational level was classified into low (primary school or lower), middle (lower and intermediate vocational training) or high (higher vocational education and university). Maternal nonverbal IQ was assessed by a computerized version of set I from the Ravens Advanced Progressive Matrices Test, which has been demonstrated to be a valid and reliable short form to assess nonverbal IQ (Chiesi, Ciancaleoni, Galli, & Primi, 2012). In addition, we measured children’s self‐reported internalizing, externalizing and attention problems at age 10 years with the Brief Problem Monitor (Achenbach, McConaughy, Ivanova, & Rescorla, 2011).

### Statistical analyses

First, the associations between psychotic experiences and school performance score were examined with multivariable linear regression analyses. Analyses were adjusted for sex, age, ethnicity, maternal educational level, maternal nonverbal IQ and whether the questionnaire on psychotic experiences was completed alone or with help from others. Second, we additionally adjusted for nonverbal IQ at age 6 years. Third, in a sensitivity step, we repeated the main analysis with additional adjustment for self‐reported co‐occurring emotional and behavioural problems at age 10 years, in order to assess whether the association between psychotic experiences and school performance might be explained by more general psychopathology. It is important to note that in a cross‐sectional analysis co‐occurring emotional and behavioural problems can be conceived as confounders, but also as mediators of the relationship between psychotic experiences and school performance. Given that emotional and behavioural problems were measured at the same time point as psychotic experiences, emotional and behavioural problems could not be studied as a mediator, and thus, statistically distinguishing between confounding and mediation is not possible.

Fourth, we performed ordinal logistic regression analysis to assess the relationship of nonverbal IQ with psychotic experiences, and separately for auditory and visual hallucinations. As a sensitivity analysis, we also assessed this relationship in the subgroup of children for whom we had school performance data available (*n = *2,362), in order to assess possible selection effects. We divided nonverbal IQ scores by 10 in order to enhance interpretation.

All statistical analyses were performed using R version 3.5.1 (R Core Team, 2015). Missing data on covariates were handled using multiple imputations in MICE 2.46 with 100 imputed data sets (van Buuren & Groothuis‐Oudshoorn, 2011). A complete‐case analysis yielded similar results as those obtained using multiple‐imputed data (data not shown).

## Results

### Study population characteristics

The characteristics of the school performance and IQ samples are described in Table 1. The samples are similar on most sociodemographic characteristics, including age, sex and prevalence of psychotic experiences. However, in the school performance sample, there are slightly more children of Dutch origin and more highly educated mothers. In our study population (i.e. school performance sample), 24% of children reported mild psychotic experiences and 7% of children reported moderate‐to‐severe psychotic experiences. Auditory and visual hallucinations were reported by 26% and 16%, respectively; 11% of children reported having both. There were no sex differences in the prevalence of psychotic experiences. Nonverbal IQ scores were moderately correlated with school performance scores (*r* = .40). Table S1 shows the classification in secondary school levels of children with (any) psychotic experiences and children without psychotic experiences, based on their CITO scores.

**Table 1:** Sample characteristics

	School performance sample (*n* = 2,362)		IQ sample (*n* = 3,737)	
	*n*		*n*	
*Child characteristics*				
Sex, % girls	2,362	52.6%	3,737	51.5%
Ethnicity, %	2,350		3,716	
Dutch		70.8%		66.8%
Other Western		8.5%		8.6%
Non‐Western		20.6%		24.6%
Auditory hallucinations, %	2,362		3,737	
No		74.2%		73.9%
A bit		20.4%		19.7%
Clearly		5.4%		6.4%
Visual hallucinations, %	2,362		3,737	
No		84.1%		83.4%
A bit		12.8%		12.9%
Clearly		3.1%		3.7%
Age at PEs assessment, mean (*SD*)	2,362	9.8 (0.3)	3,737	9.8 (0.4)
PEs assessment, % filled out alone	2,362	48.1%	3,736	44.3%
CITO test score, median (IQR), range (501–550)	2,362	541.0 (13.0)	2,066	541.0 (13.0)
Age at CITO test assessment, mean (*SD*)	2,119	11.8 (0.4)	1,983	11.9 (0.4)
Nonverbal IQ, mean (*SD*)	2,066	105.2 (14.2)	3,737	103.3 (14.5)
Age at IQ assessment, mean (*SD*)	2,222	6.1 (0.4)	3,737	6.0 (0.4)
*Maternal characteristics*				
Educational level, %	2,215		3,511	
High		67.5%		62.9%
Medium		30.6%		35.1%
Low		1.9%		2.0%
Nonverbal IQ, median (IQR)	2,177	100.0 (17.0)	3,629	100.0 (17.0)

PEs, psychotic experiences. School performance and IQ sample are overlapping (*n = *2,066).

### Psychotic experiences and school performance

In analyses adjusted for sociodemographic covariates and maternal educational level and nonverbal IQ, psychotic experiences at age 10 years were associated with poorer school performance scores at age 12 years (*B* = −0.61, 95% CI [−0.98;−0.25], *p* = .001), as well as with poorer scores on the language (*B* = −2.00, 95% CI [−3.20;−0.79], *p* = .001) and mathematics (*B* = −1.75, 95% CI [−2.99;−0.51], *p = *.006, Table 2; Model 1) subscales. These associations were attenuated, but remained, after additional adjustment for nonverbal IQ at age 6 years (total school performance: *B* = −0.51, 95% CI [−0.86;−0.16], *p* = .005); language: *B* = −1.67, 95% CI [−2.85;−0.50], *p* = .005); and mathematics: *B* = −1.33, 95% CI [−2.52;−0.14], *p* = .028, Table 2; Model 2).

**Table 2:** The association between psychotic experiences at age 10 years and school performance score at age 12 years

	Total school performance score (*n* = 2,362)			Language subscale percentile rank score (*n* = 2,028)			Math subscale percentile rank score (*n* = 2,028)		
	*B*	95% CI	*p*	*B*	95% CI	*p*	*B*	95% CI	*p*
Psychotic experiences									
Unadjusted	−0.48	[−0.89;−0.08]	.019	−1.64	[−2.98;−0.30]	.016	−1.37	[−2.72;−0.02]	.047
Model 1	−0.61	[−0.98;−0.25]	.001	−2.00	[−3.20;−0.79]	.001	−1.75	[−2.99;−0.51]	.006
Model 2	−0.51	[−0.86;−0.16]	.005	−1.67	[−2.85;−0.50]	.005	−1.33	[−2.52;−0.14]	.028
Auditory hallucinations									
Unadjusted	−0.58	[−1.21;0.05]	.071	−1.99	[−4.09;0.11]	.064	−1.91	[−4.03;0.21]	.078
Model 1	−0.66	[−1.23;−0.09]	.023	−2.13	[−4.02;−0.24]	.027	−2.01	[−3.96;−0.06]	.043
Model 2	−0.47	[−1.02;0.08]	.094	−1.49	[−3.33;0.35]	.113	−1.20	[−3.06;0.68]	.210
Visual hallucinations									
Unadjusted	−0.88	[−1.65;−0.11]	.024	−2.92	[−5.43;−0.41]	.022	−2.08	[−4.61;0.45]	.107
Model 1	−1.24	[−1.93;−0.54]	<.001	−3.98	[−6.24;−1.73]	<.001	−3.27	[−5.59;−0.95]	.006
Model 2	−1.16	[−1.83;−0.49]	<.001	−3.75	[−5.94;−1.56]	<.001	−2.97	[−5.20;−0.75]	.009

Range of total school performance score is 501–550. Range of psychotic experiences score is 0–4 and range of auditory/visual hallucinations score is 0–2. Model 1 is adjusted for sex, age, ethnicity, maternal educational level, maternal nonverbal IQ and whether the questionnaire on psychotic experienceswas completed alone or with help from others. Model 2 is additionally adjusted for nonverbal IQ at age 6 years.

### Visual and auditory hallucinations

Auditory and visual hallucinations were independently associated with lower total school performance scores, as well as with lower scores on the language and mathematics subscales (Table 2; Model 1). After additional adjustment for nonverbal IQ, visual hallucinations, but not auditory hallucinations, remained associated with total school performance scores, as well as with language and mathematics subscale scores (Table 2; Model 2).

### Severity of psychotic experiences

We also compared school performance scores of children with no, mild and moderate‐to‐severe psychotic experiences (Table S2). A trend test provided evidence of a dose–response relationship between severity of psychotic experiences and school performance scores (*p*‐trend = .003). The impairment in school performance of children with moderate‐to‐severe psychotic experiences (*B* = −1.68, 95% CI [−2.90;−0.45], *p = *.007) was more marked than the impairment of children with mild psychotic experiences (*B* = −0.60, 95% CI [−1.34;0.14], *p = *.11).

### Co‐occurring psychopathology

In sensitivity analyses adjusted for co‐occurring psychopathology (Table 3), psychotic experiences remained associated with total school performance scores after adjustment for internalizing problems (*B* = −0.40, 95% CI [−0.77;−0.03], *p = *.036) and externalizing problems (*B* = −0.40, 95% CI [−0.75;−0.04], *p = *.029). However, psychotic experiences did not remain associated with school performance after adjustment for attention problems (*B* = −0.10, 95% CI [−0.47;0.26], *p = *.57).

**Table 3:** The association between psychotic experiences at age 10 years and school performance scores at age 12 years, corrected for co‐occurring psychopathology at age 10 years

Psychotic experiences	Total school performance score		
	*B*	95% CI	*p*
Unadjusted	−0.48	[−0.89;−0.08]	.019
Model 1	−0.61	[−0.98;−0.25]	.001
Model 2	−0.51	[−0.86;−0.16]	.005
+ adjusted for internalizing problems	−0.40	[−0.77;−0.03]	.036
+ adjusted for externalizing problems	−0.40	[−0.75;−0.04]	.029
+ adjusted for attention problems	−0.10	[−0.47;0.26]	.57

Model 1 is adjusted for sex, age, ethnicity, maternal educational level, maternal nonverbal IQ and whether the questionnaire on psychotic experienceswas completed alone or with help from others. Model 2 is additionally adjusted for nonverbal IQ at age 6 years.

### Nonverbal IQ

Nonverbal IQ scores at age 6 years were not associated with psychotic experiences at age 10 years (Table S3). Likewise, sensitivity analyses in the smaller sample of children for whom school performance data were available, no association between nonverbal IQ and psychotic experiences was demonstrated. However, in this subsample, children with a higher nonverbal IQ score were slightly less likely to have auditory hallucinations (OR = 0.93, 95% CI [0.86–1.00], *p = *.04), suggesting some selection effects.

## Discussion

In this population‐based cohort study, psychotic experiences at age 10 years, as assessed by self‐reported hallucinations, were prospectively associated with poorer – albeit of small magnitude – school performance at age 12 years, including poorer language and mathematical skills. These associations were independent of sociodemographic characteristics, maternal and early childhood nonverbal IQ, as well as co‐occurring internalizing and externalizing problems. However, the association between psychotic experiences and school performance was not independent of co‐occurring attention problems. This indicates that the association with school performance may be accounted for by attention problems – or a common vulnerability underlying both psychotic experiences and attention problems – rather than be solely attributable to the effects of psychotic experiences. Nevertheless, the present findings are in line with prior work indicating that children with psychotic experiences represent a particularly vulnerable group at risk for adverse clinical and functional outcomes (Bhavsar, McGuire, MacCabe, Oliver, & Fusar‐Poli, 2018; Healy et al., 2019; Yates et al., 2018). Although the observed effect estimates were small, even minor deviations in school test performance may have important consequences for educational attainment of a small number of children.

Our results confirm previous findings of lower educational achievement in individuals with psychotic experiences (Bartels‐Velthuis et al., 2011; Davies et al., 2018; Unterrassner et al., 2017). As opposed to most previous studies, we accounted for maternal education and maternal nonverbal IQ, as well as early childhood nonverbal IQ. Accounting for IQ is important as children with lower IQ levels are more likely to have poorer school performance scores (Lynn, Meisenberg, Mikk, & Williams, 2007) and potentially at increased risk for psychotic experiences (Horwood et al., 2008). Our study also extended previous findings by independently investigating auditory and visual hallucinations, which indicated that visual hallucinations had particularly robust associations with school performance. This is in line with studies in psychosis demonstrating that visual hallucinations are associated with greater clinical impairments and a poorer prognosis (Waters et al., 2014).

It should be noted that the observed effect sizes of the relationship between psychotic experiences and school performance were small and that the confidence intervals were wide, which could be expected in population‐based studies examining subclinical psychotic phenomena (Paulus & Thompson, 2019). Moreover, in the Netherlands, since the CITO examination is used as important guidance for official recommendations concerning secondary school level (van der Lubbe, 2007), small differences in test scores have the potential to substantially impact a child’s long‐term educational trajectory. Given our observation that the CITO score of children with moderate‐to‐severe psychotic experiences was approximately 2 points lower than their nonaffected peers, and formal recommendations for a child to advance to a preuniversity school has a window of only 5 points, a difference of 2 points might conceivably influence the level to which a child is referred.

In contrast to previous studies, we did not observe an association between early childhood IQ and pre‐adolescent psychotic experiences (Horwood et al., 2008; Polanczyk et al., 2010). This might be due to our assessment of nonverbal IQ at a young age, which potentially did not capture the full spectrum of cognitive abilities. Similarly, other population‐based studies also have found weak evidence of a prospective association between IQ and psychotic experiences (Mollon, David, Zammit, Lewis, & Reichenberg, 2018; Wiles et al., 2006). Rather, IQ deficits may be a feature of clinical psychosis, whereas psychotic experiences may be associated with more subtle neuropsychological deficits, such as impaired processing speed (Kelleher, Clarke et al., 2012), which are not easily captured by more global measures such as IQ. In addition, it may be difficult to detect an effect in younger samples, in which psychotic experiences have a higher prevalence and are more likely to be transient (Kelleher, Connor et al., 2012).

Since psychotic experiences often co‐occur with psychopathology (Kelleher, Keeley et al., 2012; van Os & Reininghaus, 2016; Polanczyk et al., 2010; Wigman et al., 2012), the relationship of psychotic experiences with any outcome, including school performance, should be considered in the light of co‐occurring mental health problems. In the present study, we observed that the relationship between psychotic experiences and school performance was independent of co‐occurring internalizing and externalizing problems. However, the association was not independent of co‐occurring attention problems, which raises the question whether psychotic experiences are causally related to impaired school performance. Attention‐deficit/hyperactivity disorder (ADHD) symptoms, and in particular symptoms of inattention (rather than hyperactivity), are robustly associated with poor school performance (Daley & Birchwood, 2010; Pingault et al., 2011). In addition, ADHD symptoms and psychotic experiences often co‐occur, but it is unclear which mechanisms underlie this association (Hennig, Jaya, Koglin, & Lincoln, 2017; McGrath et al., 2016). This may indicate that attention problems – or rather, a common vulnerability underlying both psychotic experiences and attention problems – might explain the association between psychotic experiences and school performance. An alternative, but arguably less likely explanation is that attention problems constitute a mechanism through which psychotic experiences affect school performance (i.e. a mediating effect). In the case of mediation, the correction for attention problems would have resulted in over‐adjustment and therefore an underestimation of the actual effect. However, it is not possible to statistically differentiate between confounding and mediation, because attention problems and psychotic experiences were measured at the same time point. Since attention skills are in itself part of a more general construct of cognition and executive functioning (Diamond, 2013), the adjustment for attention problems should be interpreted carefully. The complex interplay between psychotic experiences, attention problems and school performance warrants further exploration in prospective designs with repeated measures to explore (causal) directionalities of effects.

There are several other, nonmutually exclusive, mechanisms that could explain the relationship between psychotic experiences and poor school performance. Psychotic experiences may impact school performance through the psychological distress resulting from having psychotic experiences. Approximately 75% of children with psychotic experiences report feeling distressed by them (Kelleher et al., 2015). Furthermore, psychotic experiences and school performance could share a common pathophysiology, such as reduced functional connectivity of higher‐order cognitive networks (Karcher, O’Brien, Kandala, & Barch, 2019). Psychotic experiences and poor school performance could also share a common aetiology, such as childhood trauma (Janssen et al., 2004; McGrath et al., 2017; Trotta, Murray, & Fisher, 2015) or genetic risk (Legge et al., 2019). A recent twin study reported that childhood psychotic experiences were associated with several negative functional outcomes in young adulthood, including lower life satisfaction, social isolation and obesity (Trotta et al., 2019), which was mainly explained by familial risk factors. This suggests that psychotic experiences may not directly cause functional difficulties. While we adjusted for several familial factors including maternal education and IQ, the role of other (unmeasured) familial confounding warrants further investigation.

Previous studies have reported lower levels of global functioning in both adults (Navarro‐Mateu et al., 2017) and children with psychotic experiences (Healy et al., 2018; Kelleher et al., 2015). Most of these studies used global assessment scales, which provide limited insight into specific areas of functioning that are impaired. Using an objective naturalistic outcome of school performance, we provide further evidence that psychotic experiences not only reflect a risk for severe outcomes in adulthood, but also for functional impairment in childhood. Given the high individual and public burden of functional impairment, longitudinal studies are needed to better understand the significance of psychotic experiences across specific domains of functioning. Future research would also benefit to examine the persistence and distress of psychotic experiences in relation to functional outcomes, although studies suggest that severe psychotic experiences share similar aetiologies as more mild or transient psychotic experiences (Zavos et al., 2014).

### Strengths and limitations

The main strengths of our study are the use of a population‐based sample and an objective and standardized assessment of school performance (CITO test). The CITO test is a reliable and objective predictor of secondary school attainment (De Boer, Bosker, & van der Werf, 2010). Another strength is that we accounted for early childhood IQ and co‐occurring emotional and behavioural problems in order to disentangle the specific effects of psychotic experiences on school performance.

However, several limitations need to be considered. First, due to the observational nature of this study, no causal inferences can be made. Second, due to selective loss to follow‐up and selective participation in the CITO linkage, children in this study were more often of Dutch nationality and had more highly educated mothers, which may have introduced selection effects. Third, nonverbal IQ was measured at age 6 years, whereas psychotic experiences and school performance were measured at age 10 and 12 years, respectively. However, IQ is moderately stable during childhood (Trzaskowski, Yang, Visscher, & Plomin, 2014) and in our study predictive of school performance scores, which supports the suitability of including nonverbal IQ at age 6 years as a covariate. Fourth, we assessed psychotic experiences using a self‐report questionnaire on hallucinations, which may have resulted in a suboptimal assessment of psychotic experiences since our assessment did not cover delusional thoughts. It has repeatedly been shown, however, that self‐reported hallucinations have the highest predictive power of clinician‐confirmed psychotic experiences (Gundersen et al., 2019; Horwood et al., 2008; Kelleher et al., 2009). In addition, the use of self‐reports may have led to an overestimation of the prevalence of psychotic experiences (Linscott & Van Os, 2013). This overestimation may be more marked in samples including younger children (as in the current study), because of a reduced ability to understand the questions. However, a recent study reported that the positive predictive value of self‐reported psychotic experiences against clinical judgement did not differ between assessment at ages 6–10 years and ages 11–14 years, suggesting that the predictive value might not increase with age (Moriyama et al., 2019). Furthermore, self‐reported psychotic experiences unconfirmed by clinical interview have previously been shown to predict future clinical psychotic symptoms and adverse psychological outcomes (Rimvall et al., 2019; van der Steen et al., 2018).

## Conclusions

We observed that psychotic experiences were associated with impaired school performance in pre‐adolescent children. This association was largely independent of early childhood IQ and co‐occurring internalizing and externalizing problems. However, the observed effects were small and not independent of co‐occurring attention problems. Given the importance of school performance for a multitude of quality of life outcomes, future longitudinal studies are needed to better understand the relevance and nature of the relationship between psychotic experiences and school performance.

## Supporting information


**Table S1.** Secondary school‐level classification corresponding to the CITO scores and comparison of children with and without psychotic experiences.
**Table S2.** The association between severity of psychotic experiences at age 10 years and school performance scores at age 12 years.
**Table S3.** The association between non‐verbal IQ at age 6 years and psychotic experiences at age 10 years.

## References

[jcpp13281-bib-0001] Achenbach, T.M. , & Edelbrock, C.S. (1987). Manual for the youth self‐report and profile. University of Vermont. Department of Psychiatry.

[jcpp13281-bib-0002] Achenbach, T.M. , McConaughy, S.H. , Ivanova, M.Y. , & Rescorla, L.A. (2011). Manual of the ASEBA Brief Problem Monitor (BPM). Burlington, VT: University of Vermont, Research Center for Children, Youth, & Families.

[jcpp13281-bib-0003] Bartels‐Velthuis, A.A. , van de Willige, G. , Jenner, J.A. , van Os, J. , & Wiersma, D. (2011). Course of auditory vocal hallucinations in childhood: 5‐year follow‐up study. The British Journal of Psychiatry, 199, 296–302. 21708881 10.1192/bjp.bp.110.086918

[jcpp13281-bib-0004] Bhavsar, V. , McGuire, P. , MacCabe, J. , Oliver, D. , & Fusar‐Poli, P. (2018). A systematic review and meta‐analysis of mental health service use in people who report psychotic experiences. Early intervention in psychiatry, 12, 275–285. 28805304 10.1111/eip.12464 PMC6001621

[jcpp13281-bib-0005] Bolhuis, K. , Koopman‐Verhoeff, M.E. , Blanken, L.M.E. , Cibrev, D. , Jaddoe, V.W.V. , Verhulst, F.C. , … & Tiemeier, H. (2018). Psychotic‐like experiences in pre‐adolescence: what precedes the antecedent symptoms of severe mental illness? Acta Psychiatrica Scandinavica, 138, 15–25. 29675994 10.1111/acps.12891

[jcpp13281-bib-0006] Chiesi, F. , Ciancaleoni, M. , Galli, S. , & Primi, C. (2012). Using the Advanced Progressive Matrices (Set I) to assess fluid ability in a short time frame: An item response theory–based analysis. Psychological assessment, 24, 892. 22449036 10.1037/a0027830

[jcpp13281-bib-0007] Daley, D. , & Birchwood, J. (2010). ADHD and academic performance: Why does ADHD impact on academic performance and what can be done to support ADHD children in the classroom? Child: Care, Health and Development, 36, 455–464. 10.1111/j.1365-2214.2009.01046.x 20074251

[jcpp13281-bib-0008] Davies, J. , Sullivan, S. , & Zammit, S. (2018). Adverse life outcomes associated with adolescent psychotic experiences and depressive symptoms. Social psychiatry and psychiatric epidemiology, 53, 497–507. 29556667 10.1007/s00127-018-1496-z PMC5908822

[jcpp13281-bib-0009] De Boer, H. , Bosker, R.J. , & van der Werf, M.P.C. (2010). Sustainability of teacher expectation bias effects on long‐term student performance. Journal of Educational Psychology, 102, 168.

[jcpp13281-bib-0010] Diamond, A. (2013). Executive functions. Annual Review of Psychology, 64, 135–168. 10.1146/annurev-psych-113011-143750 PMC4084861 23020641

[jcpp13281-bib-0011] Gundersen, S.V. , Goodman, R. , Clemmensen, L. , Rimvall, M.K. , Munkholm, A. , Rask, C.U. , … & Jeppesen, P. (2019). Concordance of child self‐reported psychotic experiences with interview‐and observer‐based psychotic experiences. Early Intervention in Psychiatry, 13, 619–626. 29516640 10.1111/eip.12547

[jcpp13281-bib-0012] Healy, C. , Brannigan, R. , Dooley, N. , Coughlan, H. , Clarke, M. , Kelleher, I. , & Cannon, M. (2019). Childhood and adolescent psychotic experiences and risk of mental disorder: a systematic review and meta‐analysis. Psychological Medicine, 49, 1589–1599. 31088578 10.1017/S0033291719000485

[jcpp13281-bib-0013] Healy, C. , Campbell, D. , Coughlan, H. , Clarke, M. , Kelleher, I. , & Cannon, M. (2018). Childhood psychotic experiences are associated with poorer global functioning throughout adolescence and into early adulthood. Acta Psychiatrica Scandinavica, 138, 26–34. 29855047 10.1111/acps.12907

[jcpp13281-bib-0014] Hennig, T. , Jaya, E.S. , Koglin, U. , & Lincoln, T.M. (2017). Associations of attention‐deficit/hyperactivity and other childhood disorders with psychotic experiences and disorders in adolescence. European Child & Adolescent Psychiatry, 26, 421–431. 27623819 10.1007/s00787-016-0904-8

[jcpp13281-bib-0015] Horwood, J. , Salvi, G. , Thomas, K. , Duffy, L. , Gunnell, D. , Hollis, C. , … & Harrison, G. (2008). IQ and non‐clinical psychotic symptoms in 12‐year‐olds: results from the ALSPAC birth cohort. The British Journal of Psychiatry, 193, 185–191. 18757973 10.1192/bjp.bp.108.051904 PMC2806573

[jcpp13281-bib-0016] Janssen, I. , Krabbendam, L. , Bak, M. , Hanssen, M. , Vollebergh, W. , de Graaf, R. , & Os, J. (2004). Childhood abuse as a risk factor for psychotic experiences. Acta Psychiatrica Scandinavica, 109, 38–45. 14674957 10.1046/j.0001-690x.2003.00217.x

[jcpp13281-bib-0017] Karcher, N.R. , O’Brien, K.J. , Kandala, S. , & Barch, D.M. (2019). Resting state functional connectivity and psychotic‐like experiences in childhood: Results from the adolescent brain cognitive development study. Biological Psychiatry, 86, 7–15. 30850130 10.1016/j.biopsych.2019.01.013 PMC6588441

[jcpp13281-bib-0018] Kaymaz, N. , Drukker, M. , Lieb, R. , Wittchen, H.U. , Werbeloff, N. , Weiser, M. , … & van Os, J. (2012). Do subthreshold psychotic experiences predict clinical outcomes in unselected non‐help‐seeking population‐based samples? A systematic review and meta‐analysis, enriched with new results. Psychological Medicine, 42, 2239–2253. 22260930 10.1017/S0033291711002911

[jcpp13281-bib-0019] Kelleher, I. , Clarke, M.C. , Rawdon, C. , Murphy, J. , & Cannon, M. (2012). Neurocognition in the extended psychosis phenotype: performance of a community sample of adolescents with psychotic symptoms on the MATRICS neurocognitive battery. Schizophrenia bulletin, 39, 1018–1026. 22927672 10.1093/schbul/sbs086 PMC3756771

[jcpp13281-bib-0020] Kelleher, I. , Connor, D. , Clarke, M.C. , Devlin, N. , Harley, M. , & Cannon, M. (2012). Prevalence of psychotic symptoms in childhood and adolescence: a systematic review and meta‐analysis of population‐based studies. Psychological Medicine, 42, 1857–1863. 22225730 10.1017/S0033291711002960

[jcpp13281-bib-0021] Kelleher, I. , Harley, M. , Murtagh, A. , & Cannon, M. (2009). Are screening instruments valid for psychotic‐like experiences? A validation study of screening questions for psychotic‐like experiences using in‐depth clinical interview. Schizophrenia Bulletin, 37, 362–369. 19542527 10.1093/schbul/sbp057 PMC3044617

[jcpp13281-bib-0022] Kelleher, I. , Keeley, H. , Corcoran, P. , Lynch, F. , Fitzpatrick, C. , Devlin, N. , … & Cannon, M. (2012). Clinicopathological significance of psychotic experiences in non‐psychotic young people: evidence from four population‐based studies. The British Journal of Psychiatry, 201, 26–32. 22500011 10.1192/bjp.bp.111.101543

[jcpp13281-bib-0023] Kelleher, I. , Wigman, J.T.W. , Harley, M. , O'Hanlon, E. , Coughlan, H. , Rawdon, C. , … & Cannon, M. (2015). Psychotic experiences in the population: Association with functioning and mental distress. Schizophrenia research, 165, 9–14. 25868930 10.1016/j.schres.2015.03.020

[jcpp13281-bib-0024] Khandaker, G.M. , Barnett, J.H. , White, I.R. , & Jones, P.B. (2011). A quantitative meta‐analysis of population‐based studies of premorbid intelligence and schizophrenia. Schizophrenia research, 132, 220–227. 21764562 10.1016/j.schres.2011.06.017 PMC3485562

[jcpp13281-bib-0025] Kooijman, M.N. , Kruithof, C.J. , van Duijn, C.M. , Duijts, L. , Franco, O.H. , van Ijzendoorn, M.H. , & Jaddoe, V.W.V. (2016). The Generation R Study: Design and cohort update 2017. European Journal of Epidemiology, 31, 1243–1264. 28070760 10.1007/s10654-016-0224-9 PMC5233749

[jcpp13281-bib-0026] Legge, S.E. , Jones, H.J. , Kendall, K.M. , Pardiñas, A.F. , Menzies, G. , Bracher‐Smith, M. , … & Walters, J.T.R. (2019). Association of genetic liability to psychotic experiences with neuropsychotic disorders and traits. JAMA Psychiatry, 76, 1256. 31553412 10.1001/jamapsychiatry.2019.2508 PMC6764002

[jcpp13281-bib-0027] Linscott, R.J. , & Van Os, J. (2013). An updated and conservative systematic review and meta‐analysis of epidemiological evidence on psychotic experiences in children and adults: On the pathway from proneness to persistence to dimensional expression across mental disorders. Psychological Medicine, 43, 1133–1149. 22850401 10.1017/S0033291712001626

[jcpp13281-bib-0028] Lynn, R. , Meisenberg, G. , Mikk, J. , & Williams, A. (2007). National IQs predict differences in scholastic achievement in 67 countries. Journal of Biosocial Science, 39, 861–874. 17381890 10.1017/S0021932007001964

[jcpp13281-bib-0029] McGrath, J.J. , McLaughlin, K.A. , Saha, S. , Aguilar‐Gaxiola, S. , Al‐Hamzawi, A. , Alonso, J. , … & Kessler, R.C. (2017). The association between childhood adversities and subsequent first onset of psychotic experiences: A cross‐national analysis of 23 998 respondents from 17 countries. Psychological Medicine, 47, 1230–1245. 28065209 10.1017/S0033291716003263 PMC5590103

[jcpp13281-bib-0030] McGrath, J.J. , Saha, S. , Al‐Hamzawi, A. , Andrade, L. , Benjet, C. , Bromet, E.J. , … & Kessler, R.C. (2016). The bidirectional associations between psychotic experiences and DSM‐IV mental disorders. American Journal of Psychiatry, 173, 997–1006. 10.1176/appi.ajp.2016.15101293 PMC5175400 26988628

[jcpp13281-bib-0031] Mollon, J. , David, A.S. , Zammit, S. , Lewis, G. , & Reichenberg, A. (2018). Course of cognitive development from infancy to early adulthood in the psychosis spectrum. JAMA Psychiatry, 75, 270–279. 29387877 10.1001/jamapsychiatry.2017.4327 PMC5885954

[jcpp13281-bib-0032] Moriyama, T.S. , van Os, J. , Gadelha, A. , Pan, P.M. , Salum, G.A. , Manfro, G.G. , … & Drukker, M. (2019). Differences between self‐reported psychotic experiences, clinically relevant psychotic experiences, and attenuated psychotic symptoms in the general population. Frontiers in Psychiatry, 10. 10.3389/fpsyt.2019.00782 PMC6829673 31736802

[jcpp13281-bib-0033] Navarro‐Mateu, F. , Alonso, J. , Lim, C.C.W. , Saha, S. , Aguilar‐Gaxiola, S. , Al‐Hamzawi, A. , … & McGrath, J.J. (2017). The association between psychotic experiences and disability: results from the WHO World Mental Health Surveys. Acta Psychiatrica Scandinavica, 136, 74–84. 28542726 10.1111/acps.12749 PMC5664954

[jcpp13281-bib-0034] Paulus, M.P. , & Thompson, W.K. (2019). The challenges and opportunities of small effects: The new normal in academic psychiatry. JAMA Psychiatry, 76, 353. 30810720 10.1001/jamapsychiatry.2018.4540

[jcpp13281-bib-0035] Pingault, J.‐B. , Tremblay, R.E. , Vitaro, F. , Carbonneau, R. , Genolini, C. , Falissard, B. , & Côté, S.M. (2011). Childhood trajectories of inattention and hyperactivity and prediction of educational attainment in early adulthood: A 16‐year longitudinal population‐based study. American Journal of Psychiatry, 168, 1164–1170. 10.1176/appi.ajp.2011.10121732 21799065

[jcpp13281-bib-0036] Polanczyk, G. , Moffitt, T.E. , Arseneault, L. , Cannon, M. , Ambler, A. , Keefe, R.S.E. , & Caspi, A. (2010). Etiological and clinical features of childhood psychotic symptoms: Results from a birth cohort. Archives of General Psychiatry, 67, 328–338. 20368509 10.1001/archgenpsychiatry.2010.14 PMC3776482

[jcpp13281-bib-0037] R Core Team . (2015). R: A language and environment for statistical computing. Available from: http://www.r‐project.org

[jcpp13281-bib-0038] Rimvall, M.K. , Gundersen, S. , Clemmensen, L. , Munkholm, A. , Larsen, J.T. , Skovgaard, A.M. , & Jeppesen, P. (2019). Evidence that self‐reported psychotic experiences in children are clinically relevant. Schizophrenia Research, 204, 415. 30121187 10.1016/j.schres.2018.08.003

[jcpp13281-bib-0039] Tellegen, P.J. , Winkel, M. , Wijnberg‐Williams, B.J. , & Laros, J.A. (2005). Snijders‐Oomen niet‐verbale intelligentietest SON‐R 2, 5–7. Amsterdam: Boom Testuitgevers.

[jcpp13281-bib-0040] Trotta, A. , Arseneault, L. , Caspi, A. , Moffitt, T.E. , Danese, A. , Pariante, C. , & Fisher, H.L. (2019). Mental health and functional outcomes in young adulthood of children with psychotic symptoms: A longitudinal cohort study. Schizophrenia Bulletin. 10.1093/schbul/sbz069 PMC7442396 31361314

[jcpp13281-bib-0041] Trotta, A. , Murray, R.M. , & Fisher, H.L. (2015). The impact of childhood adversity on the persistence of psychotic symptoms: a systematic review and meta‐analysis. Psychological Medicine, 45, 2481–2498. 25903153 10.1017/S0033291715000574

[jcpp13281-bib-0042] Trzaskowski, M. , Yang, J. , Visscher, P.M. , & Plomin, R. (2014). DNA evidence for strong genetic stability and increasing heritability of intelligence from age 7 to 12. Molecular Psychiatry, 19, 380. 23358157 10.1038/mp.2012.191 PMC3932402

[jcpp13281-bib-0043] Unterrassner, L. , Wyss, T.A. , Wotruba, D. , Ajdacic‐Gross, V. , Haker, H. , & Rössler, W. (2017). Psychotic‐like experiences at the healthy end of the psychosis continuum. Frontiers in Psychology, 8, 775. 28555120 10.3389/fpsyg.2017.00775 PMC5431212

[jcpp13281-bib-0044] Van Boxtel, H. , Engelen, R. , & de Wijs, A. (2011). Wetenschappelijke verantwoording van de Eindtoets Basisonderwijs 2010. Arnhem, the Netherlands: Cito.

[jcpp13281-bib-0045] van Buuren, S. , & Groothuis‐Oudshoorn, K. (2011). mice: Multivariate imputation by chained equations in R. Journal of Statistical Software, 45, 1–67.

[jcpp13281-bib-0046] van der Lubbe, M. (2007). The end of primary school test.

[jcpp13281-bib-0047] van der Steen, Y. , Myin‐Germeys, I. , van Nierop, M. , Ten Have, M. , de Graaf, R. , van Dorsselaer, S. , … & van Winkel, R. (2018). ‘False‐positive’ self‐reported psychotic experiences in the general population: An investigation of outcome, predictive factors and clinical relevance. Epidemiology and Psychiatric Sciences, 28, 532–543. 29656729 10.1017/S2045796018000197 PMC6998918

[jcpp13281-bib-0048] van Os, J. , & Reininghaus, U. (2016). Psychosis as a transdiagnostic and extended phenotype in the general population. World Psychiatry, 15, 118–124. 27265696 10.1002/wps.20310 PMC4911787

[jcpp13281-bib-0049] Waters, F. , Collerton, D. , Ffytche, D.H. , Jardri, R. , Pins, D. , Dudley, R. , & Larøi, F. (2014). Visual hallucinations in the psychosis spectrum and comparative information from neurodegenerative disorders and eye disease. Schizophrenia Bulletin, 40(Suppl_4), S233–S245. 24936084 10.1093/schbul/sbu036 PMC4141306

[jcpp13281-bib-0050] Wigman, J.T.W. , van Nierop, M. , Vollebergh, W.A.M. , Lieb, R. , Beesdo‐Baum, K. , Wittchen, H.‐U. , van Os, J. (2012). Evidence that psychotic symptoms are prevalent in disorders of anxiety and depression, impacting on illness onset, risk, and severity—implications for diagnosis and ultra–high risk research. Schizophrenia Bulletin, 38, 247–257. 22258882 10.1093/schbul/sbr196 PMC3283146

[jcpp13281-bib-0051] Wiles, N.J. , Zammit, S. , Bebbington, P. , Singleton, N. , Meltzer, H. , & Lewis, G. (2006). Self‐reported psychotic symptoms in the general population: Results from the longitudinal study of the British National Psychiatric Morbidity Survey. The British Journal of Psychiatry, 188, 519–526. 16738341 10.1192/bjp.bp.105.012179

[jcpp13281-bib-0052] Yates, K. , Lång, U. , Cederlöf, M. , Boland, F. , Taylor, P. , Cannon, M. , … & Kelleher, I. (2018). Association of psychotic experiences with subsequent risk of suicidal ideation, suicide attempts, and suicide deaths: A systematic review and meta‐analysis of longitudinal population studies. JAMA Psychiatry, 76, 180–189. 10.1001/jamapsychiatry.2018.3514 PMC6439738 30484818

[jcpp13281-bib-0053] Zavos, H.M. , Freeman, D. , Haworth, C.M. , McGuire, P. , Plomin, R. , Cardno, A.G. , & Ronald, A. (2014). Consistent etiology of severe, frequent psychotic experiences and milder, less frequent manifestations: A twin study of specific psychotic experiences in adolescence. JAMA Psychiatry, 71, 1049–1057. 25075799 10.1001/jamapsychiatry.2014.994 PMC4156464

