# Supplementary material for: Psychotic experiences and future school performance in childhood: a population‐based cohort study
Source: J Child Psychol Psychiatry. 2020 Jun 19;62(3):357–65. doi: 10.1111/jcpp.13281 (PMC7983885; doi:10.1111/jcpp.13281)
Supplement: Supplementary file 1 — Table S1. Secondary school‐level classification corresponding to the CITO scores and comparison of children with and without psychotic experiences. Table S2. The association between severity of psychotic experiences at age 10 years and school performance scores at age 12 years. Table S3. The association between non‐verbal IQ at age 6 years and psychotic experiences at age 10 years. [file JCPP-62-357-s001.docx]

**Supporting information – Psychotic experiences and future school performance in childhood: a population-based cohort study – by Steenkamp *et al*.**

**Table S1.** Secondary school-level classification corresponding to the CITO scores and comparison of children with and without psychotic experiences.

| CITO score | Dutch secondary school level | International standard classification of education |  | Psychotic experiences | | | | |
| --- | --- | --- | --- | --- | --- | --- | --- | --- |
|  |  |  | No | | % |  | Yes | % |
| <523 | Vmbo-b or lower | ISCED 2C (special education / labour market) | 87 | | 5.3 |  | 34 | 4.7 |
| 523-528 | Vmbo-k | ISCED 2C (labour market) | 95 | | 5.8 |  | 56 | 7.7 |
| 529-532 | Vmbo-g/t | ISCED 2B (pre-vocational education) | 133 | | 8.1 |  | 56 | 7.7 |
| 533-536 | Vmbo / Havo | ISCED 2A/2B (pre-vocational / senior general education) | 177 | | 10.8 |  | 96 | 13.3 |
| 537-540 | Havo | ISCED 2A (senior general education) | 229 | | 14.0 |  | 117 | 16.2 |
| 541-544 | Havo / Vwo | ISCED 2A (senior general / pre-university education) | 309 | | 18.9 |  | 133 | 18.4 |
| 545-550 | Vwo | ISCED 2A (pre-university education) | 608 | | 37.1 |  | 232 | 32.0 |
|  |  |  | 1638 | | 100 |  | 724 | 100 |

ISCED = International Standard Classification of Education (Statistics, 2012).

**Table S2.** The association between severity of psychotic experiences at age 10 years and school performance scores at age 12 years.

|  | Total school performance score  (*n*=2362) | | |  | | Language subscale  percentile rank score (*n*=2028) | | |  | Math subscale  percentile rank score (*n*=2028) | | |
| --- | --- | --- | --- | --- | --- | --- | --- | --- | --- | --- | --- | --- |
| Severity of PEs | *B* | 95% CI | *p* |  | *B* | | 95% CI | *p* |  | *B* | 95% CI | *p* |
| No PEs (*n=*1638) | -- | (ref) |  |  | -- | | (ref) |  |  | -- | (ref) |  |
| Mild PEs (*n=*557) |  |  |  |  |  | |  |  |  |  |  |  |
| Unadjusted | -0.36 | (-1.22; 0.49) | 0.403 |  | -2.43 | | (-5.27; 0.40) | 0.092 |  | -1.85 | (-4.71; 1.01) | 0.205 |
| Model 1 | -0.74 | (-1.51; 0.04) | 0.062 |  | -3.24 | | (-5.79; -0.69) | 0.013 |  | -2.86 | (-5.49; -0.23) | 0.033 |
| Model 2 | -0.60 | (-1.34; 0.14) | 0.112 |  | -2.66 | | (-5.14; -0.17) | 0.036 |  | -2.11 | (-4.63; 0.42) | 0.102 |
| Moderate-to-severe PEs (*n=*167) | | |  |  |  | |  |  |  |  |  |  |
| Unadjusted | -2.05 | (-3.46; -0.64) | 0.004 |  | -5.97 | | (-10.62; -1.33) | 0.012 |  | -4.45 | (-9.14; 0.23) | 0.063 |
| Model 1 | -1.92 | (-3.20; -0.64) | 0.003 |  | -5.45 | | (-9.63; -1.28) | 0.010 |  | -4.02 | (-8.32; 0.29) | 0.067 |
| Model 2 | -1.68 | (-2.90; -0.45) | 0.007 |  | -4.73 | | (-8.78; -0.67) | 0.022 |  | -3.09 | (-7.22; 1.03) | 0.142 |

*Note.* PEs = psychotic experiences. Reference category is ‘No PEs’. Range of total school performance score is 501-550. Model 1 is adjusted for sex, age, ethnicity, maternal educational level, maternal non-verbal IQ, and whether PEs questionnaire was completed alone or with help from others. Model 2 is additionally adjusted for children’s non-verbal IQ. Sample sizes of severity classifications are only shown for the *n*=2362 sample. Sample sizes for the *n=*2028 sample: No PEs (*n=*1407), mild PEs (*n*=475), and moderate-to-severe PEs (*n*=146).

**Table S3**. The association between non-verbal IQ at age 6 years and psychotic experiences at age 10 years.

| Non-verbal IQ score | Psychotic experiences  (5 levels) | | |  | Auditory hallucinations  (3 levels) | | |  | Visual hallucinations  (3 levels) | | |
| --- | --- | --- | --- | --- | --- | --- | --- | --- | --- | --- | --- |
|  | OR | 95% CI | *p* |  | OR | 95% CI | *p* |  | OR | 95% CI | *p* |
| IQ sample (*n*=3737) |  |  |  |  |  |  |  |  |  |  |  |
| Unadjusted | 1.00 | (0.95–1.04) | 0.89 |  | 0.97 | (0.92–1.02) | 0.27 |  | 1.02 | (0.96–1.08) | 0.47 |
| Model 1 | 0.98 | (0.93–1.03) | 0.37 |  | 0.96 | (0.91–1.01) | 0.09 |  | 1.00 | (0.94–1.06) | 0.98 |
| School performance sample (*n=*2362) | |  |  |  |  |  |  |  |  |  |  |
| Unadjusted | 0.97 | (0.91–1.03) | 0.35 |  | 0.94 | (0.88–1.01) | 0.08 |  | 1.01 | (0.93–1.10) | 0.81 |
| Model 1 | 0.95 | (0.89–1.02) | 0.18 |  | 0.93 | (0.86–1.00) | 0.04 |  | 0.98 | (0.90–1.07) | 0.69 |

OR per 10-unit increase in IQ score. Model 1 is adjusted for sex, age, ethnicity, maternal educational level, maternal non-verbal IQ, and whether PEs questionnaire was completed alone or with help from others.
